# Comparison of metabolic rate between two genetically distinct populations of lake sturgeon

**DOI:** 10.1002/ece3.10470

**Published:** 2023-08-30

**Authors:** Gwangseok R. Yoon, Matt J. Thorstensen, William S. Bugg, Ian A. Bouyoucos, David Deslauriers, W. Gary Anderson

**Affiliations:** ^1^ Department of Biological Sciences University of Manitoba Winnipeg Manitoba Canada; ^2^ Department of Biological Sciences University of Toronto Scarborough Toronto Ontario Canada; ^3^ Pacific Salmon Foundation Vancouver British Columbia Canada; ^4^ Institut des sciences de la mer de Rimouski Université du Québec à Rimouski Rimouski Québec Canada

**Keywords:** fish, intraspecific variation, metabolic scaling, oxygen consumption rate, population

## Abstract

Environmental temperatures differ across latitudes in the temperate zone, with relatively lower summer and fall temperatures in the north leading to a shorter growing season prior to winter. As an adaptive response, during early life stages, fish in northern latitudes may grow faster than their conspecifics in southern latitudes, which potentially manifests as different allometric relationships between body mass and metabolic rate. In the present study, we examined if population or year class had an effect on the variation of metabolic rate and metabolic scaling of age‐0 lake sturgeon (*Acipenser fulvescens*) by examining these traits in both a northern (Nelson River) and a southern (Winnipeg River) population. We compiled 6 years of data that used intermittent flow respirometry to measure metabolic rate within the first year of life for developing sturgeon that were raised in the same environment at 16°C. We then used a Bayesian modeling approach to examine the impacts of population and year class on metabolic rate and mass‐scaling of metabolic rate. Despite previous reports of genetic differences between populations, our results showed that there were no significant differences in standard metabolic rate, routine metabolic rate, maximum metabolic rate, and metabolic scaling between the two geographically separated populations at a temperature of 16°C. Our analysis implied that the lack of metabolic differences between populations could be due to family effects/parental contribution, or the rearing temperature used in the study. The present research provided insights for conservation and reintroduction strategies for these populations of lake sturgeon, which are endangered or threatened across most of their natural range.

## Graphical abstract

We demonstrated the lack of difference in metabolic rate of age‐0 lake sturgeon (*Acipenser fulvescens*) between two genetically distinct populations despite previous reports. Our analysis suggested that parental contributions or a captive environment could have overwhelmed a subtle difference in metabolic rate between populations. Our results provided insights for conservation and reintroduction programs for the species.

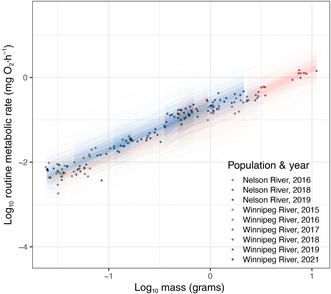

## 1 INTRODUCTION

Metabolic rate represents the rate of energy expenditure that fuels biological functions, which often indicates the metabolic costs of living in an environment. As studies have shown, metabolic rate can be repeatable and heritable (Nespolo & Franco, 2007; Norin & Malte, 2011). It is hypothesized that metabolic rate can be subject to selection, leading to intraspecific variation and thus potential transgenerational adaptation that may ultimately manifest as differential metabolic rates (Pettersen et al., 2018). Therefore, studying intraspecific variation in metabolic rate can provide insight into physiological adaptations across a range of habitats (Metcalfe et al., 2016). For example, it has been hypothesized that individuals from cold environments (i.e., higher latitudes) may have a higher metabolic rate than those from warmer environments (i.e., lower latitudes), which has been suggested as the metabolic cold adaptation hypothesis (Holeton, 1974). Research in fish has demonstrated that standard metabolic rate (SMR) may be positively correlated with latitude (White et al., 2012), which could be related to enhanced aerobic metabolism (Guderley, 2004; White et al., 2012), although the validity of this hypothesis remains debatable (Schaefer & Walters, 2010; Steffensen, 2002; Steffensen et al., 1994).

The minimum metabolic cost to maintain physiological machinery such as ion regulation, substrate cycling, and organismal integrity is SMR (Brett, 1962; Fry, 1957; Rolfe & Brown, 1997). Although SMR is relevant to understanding the indispensable energetic costs that sustain life, physiological states such as inactivity and post‐absorption may not be commonly found in the wild (Treberg et al., 2016). Thus, energetic costs that approximate routine activities such as minimal body activity and digestion may more closely represent the true metabolic costs for basal maintenance (routine metabolic rate; RMR). In contrast, the maximum metabolic rate (MMR) is the maximum energy turnover that can be achieved to maintain maximal aerobic activity (Norin & Clark, 2016). Similar to SMR, it is unlikely that fish routinely achieve MMR in the wild due to behavioral and physiological repercussions following strenuous activity (Holder et al., 2022). However, MMR is an important metric for understanding the maximal capacity of an animal that could take up oxygen from the ambient environment, which would ultimately set the biological ceiling of aerobic metabolism.

Metabolic rate does not vary independently; rather, it is integrated through genetic, developmental, morphological, behavioral, physiological, and functional processes (Clark et al., 2013; Norin & Clark, 2016; Rosenfeld et al., 2015), all of which could be selected upon by environmental variation. For example, an elevated growth rate during early life history may be an evolutionary adaptation (Arendt, 1997; Norin, 2022), as body size plays an important role in early predation and survival (Dmitriew, 2011). Further, it is hypothesized that these physiological traits, which are related to growth rate (including metabolic rate), may also be strongly subjected to co‐selection (Ricklefs & Wikelski, 2002; White et al., 2022). Previous research has demonstrated that these metabolic rate phenotypes may be closely linked to other physiological phenotypes of fish (Metcalfe et al., 2016, 2023). In the common minnow (*Phoxinus phoxinus*), individuals with accelerated growth rates following food deprivation and ad libitum feeding (i.e., compensatory growth) showed increased SMR with no change in MMR (Killen, 2014), suggesting that the growth trajectory may be closely related to variations in the energetic cost of metabolic machinery needed for maintenance (Norin, 2022; Salin et al., 2016). For example, a higher growth rate can be achieved by increased foraging activity and aggressive behaviors (Biro & Post, 2008), which could be linked to increased metabolic costs for basal maintenance (Rosenfeld et al., 2015). It is known that variation in MMR is associated with biophysical properties of the cardiorespiratory system (Hillman et al., 2013), which may be the result of physiological adaptations to local environments for foraging strategies and habitats (Killen et al., 2016).

A previous study documented high mortality during early life stages in lake sturgeon (*Acipenser fulvescens*), which was estimated to be over 90% (Caroffino et al., 2010). This implies the potential for high selective pressures on metabolic rate phenotypes during development, especially for geographically distinct populations that are exposed to drastically different thermal environments in the northern temperate zone, where the growing season is limited compared to more southerly areas prior to overwintering. As regulation of metabolic machinery can directly influence energetic allocation toward growth in fish and ultimately survival, we may expect differential selection on metabolic traits, which would promote phenotypic differences across populations of lake sturgeon from these disparate environments (Post & Parkinson, 2001; Rosenfeld et al., 2015).

Lake sturgeon is a freshwater fish species considered a living fossil, having separated from other sturgeon species tens of millions of years ago (Scott & Crossman, 1973; Shen et al., 2020). This species occupies an expansive natural latitudinal range from the Mississippi River drainage to the Hudson Bay (Ilves & Randall, 2007; Peterson et al., 2007). For populations within Manitoba, Canada, genetic studies have identified distinct populations consistent with natural geomorphological barriers separated by approximately 600 km (3 degrees), which impede movement between northern and southern groups (Ferguson & Duckworth, 1997; Kjartanson et al., 2023). Following the spawning, the average temperature during the major growing season (June to September) for the southern and northern populations is 18.6 ± 2.9 and 13.9 ± 2.9°C, respectively. Thus, it is possible that there may be allometric differences in metabolic rate between these two populations. Indeed, previous work demonstrated that local adaptation may be a source of variation in metabolic rate in age‐0 lake sturgeon (Bugg et al., 2020). When raised at the same temperature in a common garden design, individuals from a northern population showed lower RMR with enhanced growth rates compared to individuals from a southern population (Bugg et al., 2020). This finding is particularly interesting because the population difference in growth performance seems to be a result of potential countergradient variation to compensate for a shorter growing season at the higher latitudes (Pettersen, 2020; Pettersen et al., 2018). Thus, understanding intraspecific variation in metabolic rate of geographically distinct populations of lake sturgeon during early life facilitates the interpretation of adaptive phenotypic responses at a vulnerable life stage (Dmitriew, 2011; Killen et al., 2007; Pettersen et al., 2018; Speakman, 2005).

In the present study, we tested the hypothesis that variation in metabolic rate would be correlated with local adaptation and/or lifestyle between two populations (Killen et al., 2016), as well as potential differences across years. We predicted that the relationship between metabolic rate and body mass ($\dot{M}O_{2}=a\times {\text{Body mass}}^{b}$) between two populations would be different and metabolic scaling (*b*) would be higher in the northern population. In this context, we congregated metabolic rate data that were generated annually for the last 6 years in juvenile lake sturgeon from the same experimenter and using the same methodology, facilitating a robust examination of intraspecific variation across populations and year classes. This dataset is particularly useful as it becomes possible to mitigate the effect of experimental artifacts because of the use of consistent respirometry methodology (Chabot et al., 2016; Killen et al., 2021; Raby et al., 2020).

## 2 MATERIALS AND METHODS

### 2.1 Animal husbandry

As the environment during early life could significantly influence phenotypic development in lake sturgeon (Dammerman et al., 2015), metabolic rate data in this analysis were used from age‐0 lake sturgeon that were raised under the same environmental conditions of temperature (16°C), dissolved oxygen (100%), and without substrate in rearing tanks. The data analyzed here include previously published and unpublished data (Bugg et al., 2020, 2023; Yoon et al., 2020, 2021; Yoon, Amjad, et al., 2022; Yoon, Bugg, et al., 2022; Yoon, Deslauriers, & Anderson, 2019; Yoon, Deslauriers, Enders, et al., 2019; Yoon, Groening, et al., 2022; Yoon, Laluk, et al., 2022).

**FIGURE 1:**
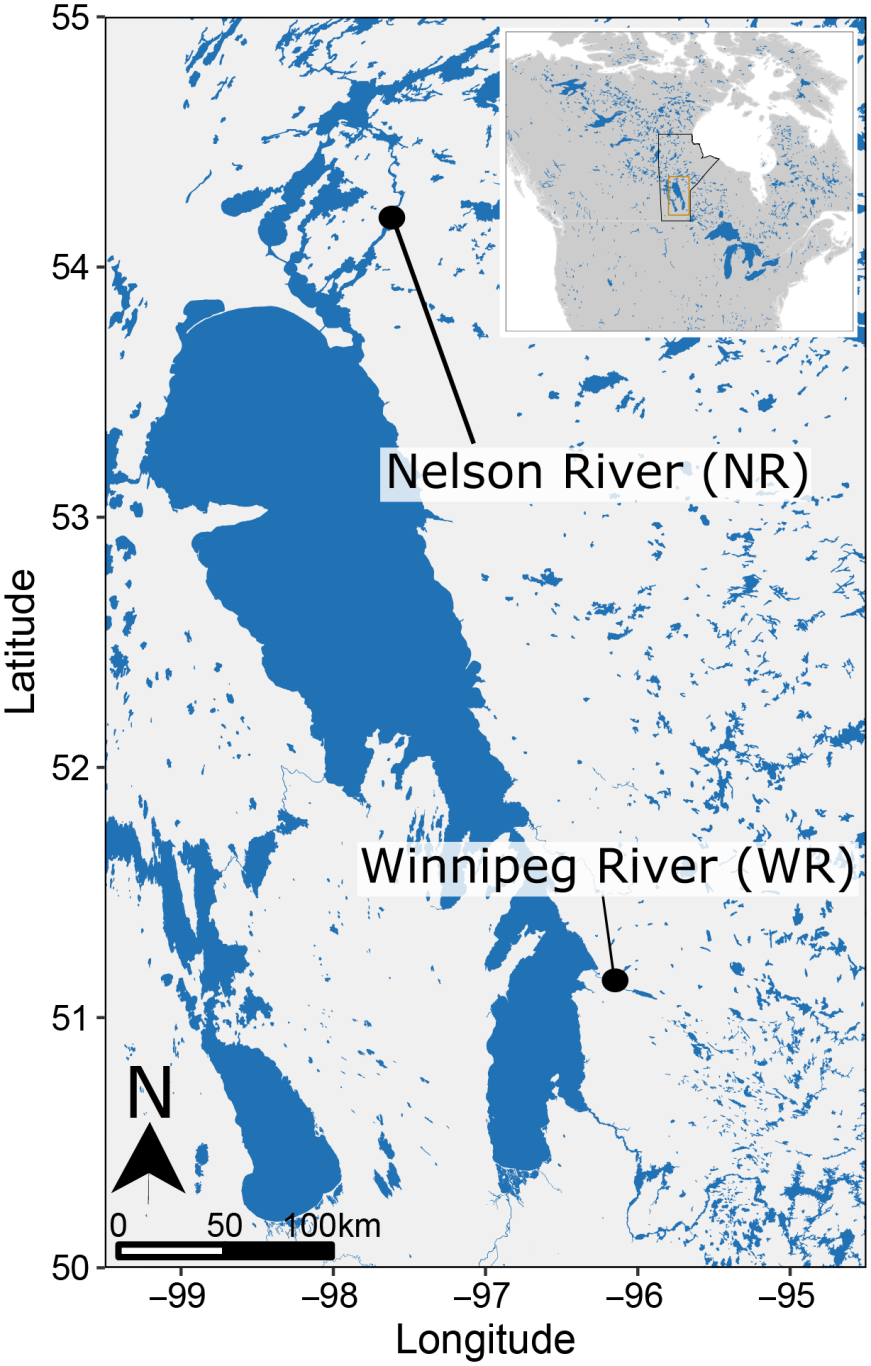
Map of collection sites for the parents of lake sturgeon (*Acipenser fulvescens*) used in the present study. The Nelson River represents the northern population used, while the Winnipeg River represents a more southern population.

In brief, the protocol for collection of gametes was performed as previously described (Boase et al., 2011; Genz et al., 2014) at either the Winnipeg River (50°17′52″ N, 95°32′51″ W; representing the southern population) or the Nelson River (55°19′36″ N, 96°54′16″ W; representing the northern population) (Figure 1). Following collection, gametes were then transferred to the Animal Holding Facility at the University of Manitoba or the Grand Rapid Fish Hatchery, where fish were raised until the end of experimentation. Upon arrival, eggs were fertilized, then submerged in a clay solution made from Fuller's earth and gently stirred by hand for 1 h to remove adhesiveness, following which all embryos were raised under the same environmental conditions. When yolk absorption was near complete, freshly hatched *Artemia* spp. nauplii were introduced to enhance prey recognition and transition fish to exogenous food sources. At the onset of exogenous feeding, *Artemia* were provided ad libitum three times a day. After approximately 3 weeks of feeding on *Artemia*, fish were gradually transitioned to a bloodworm diet. As flowthrough aquaria were used to rear developing sturgeon, ammonia, nitrite, and nitrate levels were found to be negligible throughout the studies.

### 2.2 Measurement of metabolic rate

All animals used in these studies were reared and sampled in compliance with the animal use and care guidelines established by the Canadian Council for Animal Care and approved by the Animal Care Committee at the University of Manitoba (#F015‐007 and F019‐006).

Oxygen uptake rate ($\dot{M}O_{2}$, in mg O_2_ h^−1^; a proxy for metabolic rate) was assessed using intermittent flow respirometry following the previously published protocol with modifications where applicable (Yoon et al., 2021). We used three sizes of metabolic chambers to accommodate age‐0 fish with a range of body mass throughout the studies (0.0157–11.09 g). The chamber volume and tubing volume for each size were 3.36 ± 0.15 mL (mean ± SD) with 1.54 ± 0.01 mL, 42.32 ± 1.06 with 3.07 ± 0.11, and 642.58 ± 13.36 with 67.98 ± 15.72 mL. The average ratio between body mass and chamber volume was 74.59 ± 51.08.

The respirometry equipment consisted of borosilicate chambers with oxygen sensor spots (PreSens) in a water bath, to which fiber optic cables were connected to a Witrox 4 Oxygen meter. Air saturation levels of dissolved oxygen were recorded at 1 Hz. In brief, fish were haphazardly captured from rearing tanks and placed into metabolic chambers. The flow within the chambers was enough to fully replenish oxygen concentration levels, but low enough to not cause any physical disturbance to the fish. A black curtain was hung around the respirometry setup to minimize the visual disturbance, while dimmed light was provided during the daytime to minimize the influence of the photoperiod used for rearing fish (12 L:12 D). The measurement of $\dot{M}O_{2}$ lasted for 6 h or 24 h depending on the objective of each study. Then, a standardized exercise protocol was performed to induce MMR. As it was challenging to remove and return fish to the chambers due to fragility at an early life stage (≦55 days post hatch), we chose to exercise the fish to exhaustion by initiating swimming inside the metabolic chambers with increased flow for 15 min. Any fish older than 55 days post hatch were removed from the chambers and chased by gentle prodding to the tail for 15 min. Once the exercise protocol was completed, the fish were immediately returned to the chambers, and oxygen consumption was measured for three measurement cycles.

Only $\dot{M}O_{2}$ slopes with *R* ^2^ > 0.9 were used for data analysis. SMR was estimated from the 20th quantile excluding the first 6 h of acclimation (Chabot et al., 2016), while RMR was recorded as the average of $\dot{M}O_{2}$ excluding the first 4 or 6 h of acclimation, depending on the duration of each study. MMR was the highest $\dot{M}O_{2}$ following the standardized exercise protocol. Background respiration was measured before and after each $\dot{M}O_{2}$ trial, and the data were linearly interpolated to correct all $\dot{M}O_{2}$ data points (Rodgers et al., 2016).

### 2.3 Statistical analysis

Bayesian models were fit in *brms* v.2.18.0. For each of SMR, RMR, and MMR, log_10_ body mass was set to interact with both population and year. The data source for each model was included as a random intercept. The two populations used were from the Winnipeg River from 2015, 2016, 2017, 2018, 2019, and 2021, and the Nelson River from 2016, 2018, and 2019. Full models were written as the following:

$$\begin{array}{c} {\mathrm{Log}}_{10}\;\text{Metabolic rate}={\mathrm{Log}}_{10}\;\text{Body mass}\times \text{Population}\times \text{Year}\\ \;+\left(1\right|\;\text{Data Source}) \end{array}$$

where Metabolic rate is either SMR, RMR, or MMR, Body mass is the body mass of fish (g), Population is southern or northern, Year is the year class of the fish. Data source is dataset used for analysis from each year.

In addition, we scaled metabolic rate to a standardized body mass to account for the variation of mass between experimental groups following the method (Yoon, Laluk, et al., 2022).

$$\dot{M}O_{2\;\text{scaled}}=\dot{M}O_{2\;\text{measured}}\times {\left(M\times {M_{s}}^{-1}\right)}^{1–b},$$

where $\dot{M}O_{2\;\text{scaled}}$ is the scaled metabolic rate, $\dot{M}O_{2\;\text{measured}}$ is measured $\dot{M}O_{2}$, *M* is the measured body mass (g), $M_{s}$ is the body mass to be scaled (i.e., 1 g), and b is the mass‐scaling exponent (0.89) (Jerde et al., 2019). The models of scaled metabolic rate were similar to the other models of metabolic rate, except log_10_ body mass was removed from independent variables in the model formulae to avoid conditioning on a response variable.

While a mixed effects modeling framework using random effects for slopes and intercepts in years and population effects on metabolic rate may have been effective, we opted to use an interaction‐based approach with fixed effects because of the few groups within the potential random effects terms (two populations, 3 years within one of the populations) (Arnqvist, 2020; Gomes, 2022; Harrison et al., 2018). Moreover, inferences about the effects of year within the population and of populations overall were desired, and the few groups available within the terms would have made estimations of variance difficult (Gomes, 2022). Thus, a fixed‐effects approach was taken for comparing across both years and populations. A Gaussian distribution was used for fitting each model. Four Hamiltonian Monte Carlo Markov Chains were used over 5000 warmup iterations and 15,000 sampling iterations each (60,000 sampling iterations total per model). A relatively uninformative prior for fixed effects was set with mean 0 and standard deviation 1 for models of metabolic rate, and mean 0 and standard deviation 100 for models of scaled metabolic rate. Model fit was assessed with the potential scale reduction statistic *R̂*, posterior predictive checks, and trace plots. No divergent transitions were observed during model sampling.

The R package *emmeans* v1.8.1–1 was used to test for differences in marginal means (i.e., metabolic rates) and trends (i.e., metabolic mass scaling) between populations and between years within populations for each model. 95% highest posterior density (HPD) intervals were used to contrast populations and years within populations. The R packages *tidybayes* v3.0.2, *tidyverse* v1.3.2, and *modelr* v0.1.9 were used to visualize model results. All data analyses were performed in R 4.2.2 (R Core Team, 2022).

## 3 RESULTS

A total of *n* = 165 individuals were included in the dataset overall, from 11 different data sources (between 7 and 61 individuals from each data source) (Table S1). While the full dataset was used in the RMR and MMR models, in the SMR model, data were available from *n* = 69 individuals from seven data sources (between 7 and 14 individuals from each data source).

**FIGURE 2:**
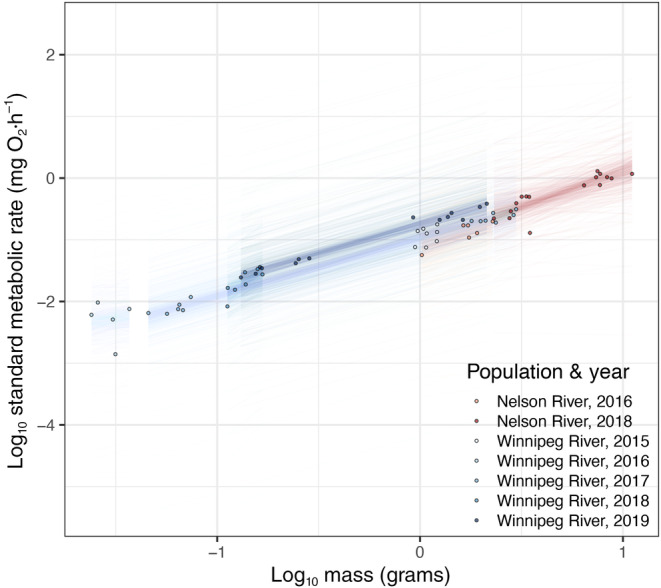
Bayesian model of log_10_ standard metabolic rate and the interaction of log_10_ body mass, population, and year. Points represent raw data used for model fitting, while lines represent 500 draws from the expectation of the posterior distribution of the model fit to the data.

In the SMR model (Figure 2), estimated marginal means and trends did not differ between the Nelson and Winnipeg River populations overall (Table S2). Between 2016 and 2018 and between populations, no difference was observed between estimated marginal means (Figure S2). Estimated marginal trends did not differ between years within either river (Table S3).

**FIGURE 3:**
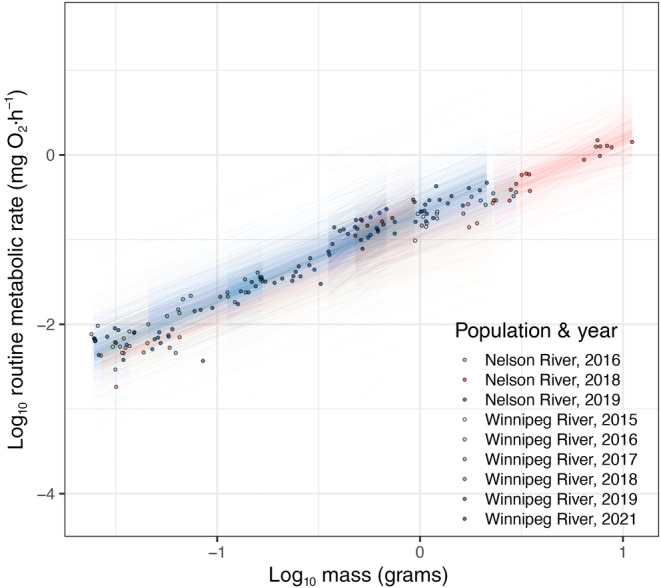
Bayesian model of log_10_ routine metabolic rate and the interaction of log_10_ body mass, population, and year. Points represent raw data used for model fitting, while lines represent 500 draws from the expectation of the posterior distribution of the model fit to the data.

In the RMR model (Figure 3), estimated marginal means and trends did not differ between the two populations (Table S4). Between years within populations, no difference was observed in estimated marginal means (Table S5). Estimated marginal trends were not different between any years in the Nelson River (Table S5). In the Winnipeg River 2017, had a lower slope than 2019 (estimate −0.201, −0.303 to −0.097 95% HPD) (Table S5).

**FIGURE 4:**
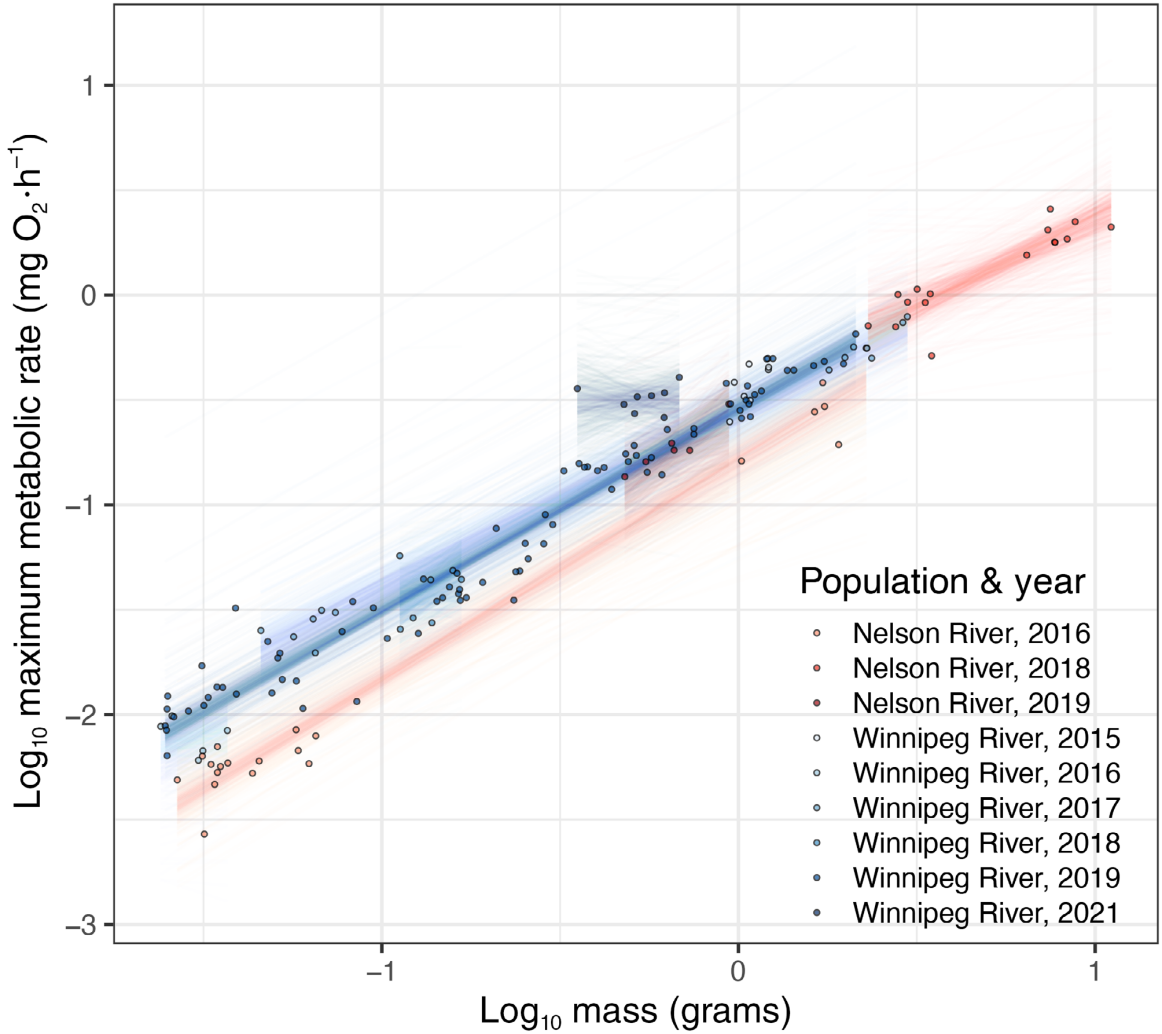
Bayesian model of log_10_ maximum metabolic rate and the interaction of log_10_ body mass, population, and year. Points represent raw data used for model fitting, while lines represent 500 draws from the expectation of the posterior distribution of the model fit to the data.

In the MMR model (Figure 4), estimated marginal means and trends did not differ between the two populations overall (Table S6). In the Winnipeg River, 2016 was lower than 2021 (estimate −1.204, −2.241 to −0.190 95% HPD), 2017 was lower than 2021 (−0.463, −0.839 to −0.109 95% HPD), 2018 was lower than 2021 (estimate −0.637, −1.155 to −0.099 95% HPD), and 2019 was lower than 2021 (estimate −0.504, −0.829 to −0.188 95% HPD) (Table S7). These differences may be attributed to 2021 having an unusually high estimated marginal mean MMR in the Winnipeg River. Estimated marginal trends did not differ between years in the Nelson River (Table S7). However, in the Winnipeg River, estimated marginal trends were lower in 2017 than 2019 (estimate −0.112, −0.202 to −0.022 95% HPD) (Table S7). Together, these results suggest slight differences in MMR across years (in both marginal means and trends) than other analyzed metabolic metrics. Last, population did not influence scaled metabolic rate in age‐0 lake sturgeon, as all HPD intervals overlapped among groups. However, the year affected only scaled MMR in the Winnipeg River, in which the estimated marginal mean MMR was higher than the other years (Figures S1–S3).

## 4 DISCUSSION

With the present results, we demonstrated that there was no substantial difference in metabolic rates, metabolic scaling, and scaled metabolic rate between southern and northern populations despite the distinct genetic differences between these two Manitoba lake sturgeon populations (Gosselin et al., 2015; Kjartanson et al., 2023). Our analysis suggested that other factors, such as the maternal /parental effects or the rearing temperature used in the study, may have overwhelmed any potential population‐level effects on metabolic rate.

### 4.1 Metabolic rates by population

These results were surprising because in the temperate zone, thermal profiles at higher latitudes can be very different from those at lower latitudes, which could significantly influence development and growth in fish. For example, the metabolic cold adaptation hypothesis predicts that individuals inhabiting higher latitudes may grow faster than those in lower latitudes, which may be correlated to a higher metabolic rate (Guderley, 2004; White et al., 2012). A higher metabolic rate could be a result of physiological adaptations to cold temperatures in more northern latitudes or a reflection of increased growth prior to overwintering due to a shorter summer and fall that would limit growing seasons for fish (David et al., 2015; Schultz & Conover, 1997; Suzuki et al., 2010). This concept is particularly important for young of the year fish, where they must grow and allocate surplus energy to lipid reserves before the first winter of life (Biro et al., 2021).

The two populations in Manitoba are latitudinally separated by approximately 600 km (3 degrees) with historical natural barriers, which impede the movement of sturgeon and likely limit gene flow (McDougall et al., 2017). Further, the difference in annual cumulative thermal units between the two populations is approximately 675.1°C. In terms of temperature difference in the growing season, temperatures for the southern Winnipeg River population from June to September are 4.7°C higher and from October to December are 3.6°C higher than the northern Nelson River population (Bugg et al., 2021). Thus, it was plausible to expect allometric differences in metabolic rate and metabolic scaling that are consistent with environmental differences (Beachum et al., 2020; Burton et al., 2011; Healy et al., 2019; Pettersen et al., 2016).

We previously reported the diverging physiological responses of age‐0 lake sturgeon from two populations with respect to increasing environmental temperatures, such as growth, thermal tolerance, and transcriptional responses (Bugg et al., 2020). When fish were raised at 16°C, thermal tolerance (critical thermal maximum) was not different between two populations. However, when raised at increased temperatures of 20 and 24°C, fish progeny originating from the northern population consistently showed lower thermal tolerance than those from the southern population. In the same experiment, fish from the northern population showed a higher hepatosomatic index, which is a physiological indicator for energy storage, at 16°C and under increasing temperatures. Thus, it is reasonable to expect that energy allocation toward assimilation and storage in response to temperatures is different between two populations of lake sturgeon in Manitoba.

However, given the divergent evolutionary histories and wide range of habitats of the two populations studied here, the extensive phenotypic plasticity apparent in lake sturgeon may have mitigated the effects of selection on metabolic rate (Price et al., 2003; Thorstensen et al., 2021). In addition, effective population sizes of either lake sturgeon population are unknown, so small effective sizes may have led to genetic drift, which may have been exacerbated by recent substantial declines in population abundance that overwhelmed selective forces in allele frequency changes (Lande, 1976). Thus, younger individuals, as were studied in the present analyses, may have been previously selected for greater plasticity (Pottier et al., 2022).

It is hypothesized that the temperature used in the present study (16°C) is thermally neutral for aerobic metabolism in this species, which did not reveal a diverging response in metabolic rates between the two populations (Bugg et al., 2020). Also, the low effective population sizes of the lake sturgeon in Manitoba may have reduced the ability of natural selection to shift metabolic rates as an evolutionarily adaptive response to different environments. It is also possible that metabolic rate plasticity may have mitigated the effect of selection from different environmental conditions (Norin & Metcalfe, 2019). With a genetic framework, we developed several alternative hypotheses as a tool to increase clarity and maximize opportunities to address natural mechanisms in our study (Betts et al., 2021).

Population‐level genetic differentiation may not be directly related to aerobic metabolism at the temperature used in the study. For example, previous work using microsatellites or single nucleotide polymorphisms revealed differentiation among groups of lake sturgeon in Manitoba, including within the Nelson River (Gosselin et al., 2015; Kjartanson et al., 2023). However, these population genetic studies relied on neutral or nearly neutral variation and thus do not necessarily predict transcriptional differences in key mechanisms that underlie aerobic metabolism, although variability of metabolic rate seems to have a polygenic basis (Pettersen et al., 2018).

### 4.2 Impacts of interannual variation

Interannual variation may have affected the present results, as we observed a significant population effect on RMR and MMR when year class was not considered as fixed effect. In our analysis, we addressed the potential influences of year class and population on measuring metabolic rate. However, it is hypothesized that sources of interannual variation in the present study may stem from family effects across years (Dammerman et al., 2015, 2016). In line with this finding, another study in our lab demonstrated a strong maternal influence on the SMR of age‐0 lake sturgeon within the northern population (Deslauriers et al., 2023). Aerobic metabolism is closely related to mitochondrial performance, which could be heritable maternally through mitochondrial DNA (Healy et al., 2019). It was suggested that mitochondrial genotype played a role in the latitudinal variation of RMR in Atlantic killifish (*Fundulus heteroclitus*) (Healy et al., 2019). Thus, maternal influence may mask subtle differences in metabolic rate between the populations.

### 4.3 Potential sources of variation on metabolic phenotypes

Although there may be differences in transcriptional responses and growth in age‐0 lake sturgeon between two populations (Bugg et al., 2020, 2021), the ontogenetic development of metabolic rate in age‐0 lake sturgeon may be developmentally canalized due to the captive rearing conditions. It is well known that captive environments could have larger effects than genotype in salmonids (Chittenden et al., 2010). A previous study reported no difference in metabolic rates of wooly sculpin (*Clinocottus analis*) between northern and southern populations and discussed that the lack of difference could be attributed to fish becoming acclimated to the captive rearing environment (Rangel & Johnson, 2019). Thus, it is possible that captive rearing environments rather than natural environments during early life stages could have greatly negated the population effects on phenotypic development and affected the results (Burggren, 2020).

Also, a subtle difference in the environmental conditions, such as water chemistry, might have impacted early development in age‐0 lake sturgeon (Loeppky & Anderson, 2021). For instance, fish from the northern population were raised with well water or dechlorinated tap water, but those from the southern population were exclusively raised with dechlorinated tap water. Although well water was fully oxygenated prior to rearing tanks, mineral content may have been different between water sources, which may have affected our results (Deslauriers et al., 2018).

Our results demonstrated that scaled metabolic rate did not differ between two populations, which is consistent with our results of mass‐specific metabolic rate and metabolic scaling. However, it is possible that the variation in ages among groups could have affected the results across year classes, as substantial variation in metabolic rate is often associated with developmental stages during early life history in fish (Rosenfeld et al., 2015; Yagi & Oikawa, 2014). For instance, a previous study showed that ontogenetic changes in metabolic rate were associated with a dietary shift in age‐0 lake sturgeon in which a diet transition from *Artemia* to bloodworm resulted in a temporal cessation of growth with elevated RMR (Yoon, Laluk, et al., 2022). In addition, our study was focused on two populations in Manitoba, which are in the northern range of the species, while the lake sturgeon range is known to extend as far as south as northern Mississippi and Alabama (Bruch et al., 2016). A previous study showed that thermal tolerance of lake sturgeon in Wisconsin, USA, is similar to that of those from Manitoba, possibly due to their closely related thermal regimes (Wilkes, 2011; Yoon, Deslauriers, & Anderson, 2019). However, a future study would benefit from including samples of populations at more southern latitudes and standardized ages, such as 2 weeks following hatch or 2 weeks after transitioning to a bloodworm diet, to address sources of variation in metabolic rate data.

While our sample sizes may not be sufficient to describe differences between populations due to logistical constraints and limited availability of fish, the interpretation of a lack of difference in metabolic rate between two populations should be taken cautiously as metabolic responses could still be different. It is reasonable to think that developmental patterns at the natural temperature regime could be different from those raised under a constant temperature throughout the early life stages. Thus, it is less than ideal to examine population effects on metabolic rate without providing natural temperature regimes. This is particularly important because a number of studies have indicated that the environment during early life could have prolonged effects on behavior and physiology in fish, which has significant implications for conservation reintroduction programs (Anderson et al., 2022; Jonsson & Jonsson, 2014).

### 4.4 Implications for conservation and reintroduction programs

The limited success of current conservation programs for lake sturgeon has been attributed to high mortality during the first winter of life following the release of fingerlings in the fall (McDougall et al., 2014), so the importance of phenotypic development during early life on overwintering survival has been highlighted. Although the common rearing practice at conservation fish hatcheries prior to release in Manitoba employs a constant temperature (16°C or 20°C) to facilitate management, the success of the conservation program could be remarkably improved by employing a natural temperature regime at these hatcheries.

There is strong evidence from previous studies in lake sturgeon suggesting that thermal history during early life could affect metabolic responses later in life, which could ultimately influence fitness and survival. For instance, when lake sturgeon were raised at constant temperatures throughout early development, they showed significantly reduced metabolic rate and body mass after a simulated wintering event of 45 days fasting at 3.5°C (Yoon, Deslauriers, Enders, et al., 2019). However, when raised at a natural temperature regime, fish showed slightly reduced body mass but not metabolic rate following the wintering event (Yoon, Deslauriers, & Anderson, 2019). Further, when fish were raised at 20°C for 2 months and subsequently raised at 16°C for 2 months, they showed a reduced condition factor with no difference in metabolic rates in comparison to those raised at the constant temperature of 16°C; however, when challenged to a cold temperature of 3.5°C, those raised at 20°C demonstrated reduced transcriptional responses to fatty acid desaturation, which is a key response in cold acclimation (Yoon, Bugg, et al., 2022). While these studies collectively suggest long‐term effects of temperatures on physiological responses, a natural temperature regime during early development could help naturalize phenotypic responses to overwintering and could increase the post‐release survival rate.

## 5 CONCLUSION

Our research has identified factors that could potentially surmount the population effects on metabolic rate in age‐0 lake sturgeon, such as maternal effects and a captive environment, which highlights the importance of parental effects and the environment during early life on metabolic rate and growth. We propose future studies to raise fish at a natural temperature regime in a natural environment (e.g., a streamside rearing facility) or measure field metabolic rate to compare allometric differences in metabolic rate between two populations. Our improved understanding of metabolic differences between latitudinally distributed populations will help tailor conservation hatchery rearing protocols and promote enhancement of conservation efforts for lake sturgeon, a species at risk across most of its natural range.

## AUTHOR CONTRIBUTIONS

**Gwangseok R. Yoon:** Conceptualization (lead); data curation (lead); formal analysis (lead); funding acquisition (lead); investigation (lead); methodology (lead); resources (lead); validation (lead); writing – original draft (lead); writing – review and editing (lead). **Matt J. Thorstensen:** Data curation (supporting); formal analysis (supporting); investigation (supporting); methodology (supporting); resources (supporting); validation (supporting); writing – review and editing (supporting). **William S. Bugg:** Data curation (supporting); formal analysis (supporting); investigation (supporting); validation (supporting); writing – review and editing (supporting). **Ian A. Bouyoucos:** Conceptualization (supporting); data curation (supporting); formal analysis (supporting); investigation (supporting); validation (supporting); writing – review and editing (supporting). **David Deslauriers:** Data curation (supporting); formal analysis (supporting); investigation (supporting); validation (supporting); writing – review and editing (supporting). **W. Gary Anderson:** Conceptualization (supporting); formal analysis (supporting); funding acquisition (lead); investigation (supporting); supervision (lead); validation (supporting); writing – review and editing (supporting).

## FUNDING INFORMATION

This research was supported by University of Manitoba Graduate Fellowship and Sixth Prairie Conservation Fellowship awarded to G.R.Y, and Natural Sciences and Engineering Research Council/Manitoba Hydro Industrial Research Chair awarded to W.G.A.

## CONFLICT OF INTEREST STATEMENT

The authors declared that there was no competing interest.

## Supporting information


Tables S1‐S7

Figures S1‐S3

Data S1

## Data Availability

The data used to generate figures and tables are available as [Link], [Link], [Link].
