# Supplementary material for: Comparison of metabolic rate between two genetically distinct populations of lake sturgeon
Source: Ecol Evol. 2023 Aug 30;13(9):e10470. doi: 10.1002/ece3.10470 (PMC10468615; doi:10.1002/ece3.10470)
Supplement: Supplementary file 2 — Figures S1‐S3 [file ECE3-13-e10470-s002.docx]

**<Supplementary Figures>**

Comparison of metabolic rate between two genetically distinct populations of lake sturgeon

Gwangseok R. Yoon^1,2*^, Matt J. Thorstensen^1^, William S. Bugg^1.3^, Ian A. Bouyoucos^1^, David Deslauriers^4^, and W. Gary Anderson^1^

^1^Department of Biological Sciences, University of Manitoba, Winnipeg, Canada

^2^Department of Biological Sciences, University of Toronto Scarborough, Toronto, Canada

^3^ Pacific Salmon Foundation, Vancouver, BC, Canada

^4﻿^Institut des sciences de la mer de Rimouski, Université du Québec à Rimouski, Rimouski, Québec, Canada

*Corresponding author email: Gwangseok.Yoon@utoronto.ca

Keywords: intraspecific variation, population, metabolic scaling, oxygen consumption rate, fish


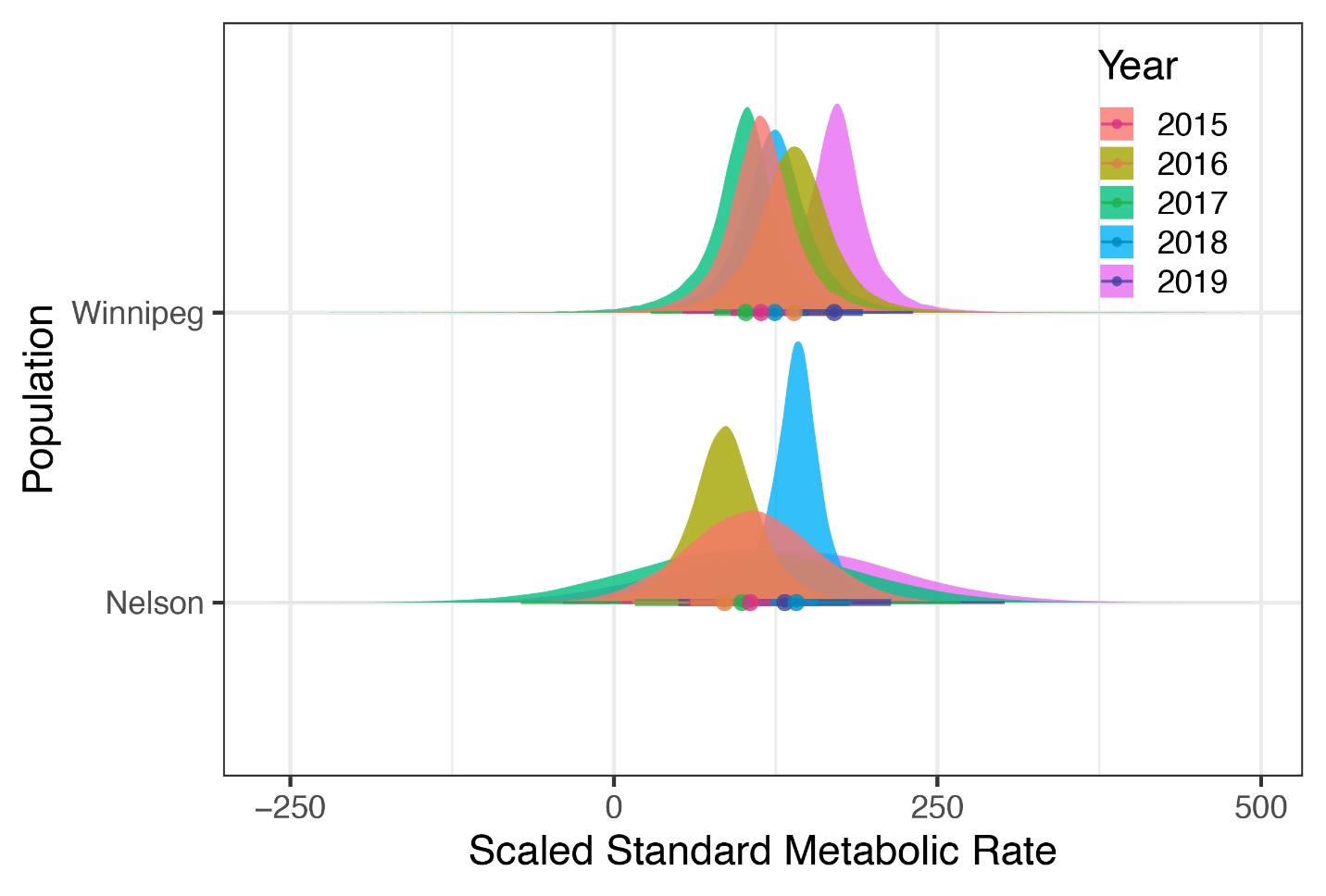


Figure S1. Posterior distributions of scaled standard metabolic rate (mgO_2_Kg^-0.89^Hr^-1^) of age-0 lake sturgeon (*Acipenser fulvescens*) between Winnipeg (southern) and Nelson (northern) population by years. 95% and 66% credible intervals are shown by the thin and bold lines, respectively, below each distribution.


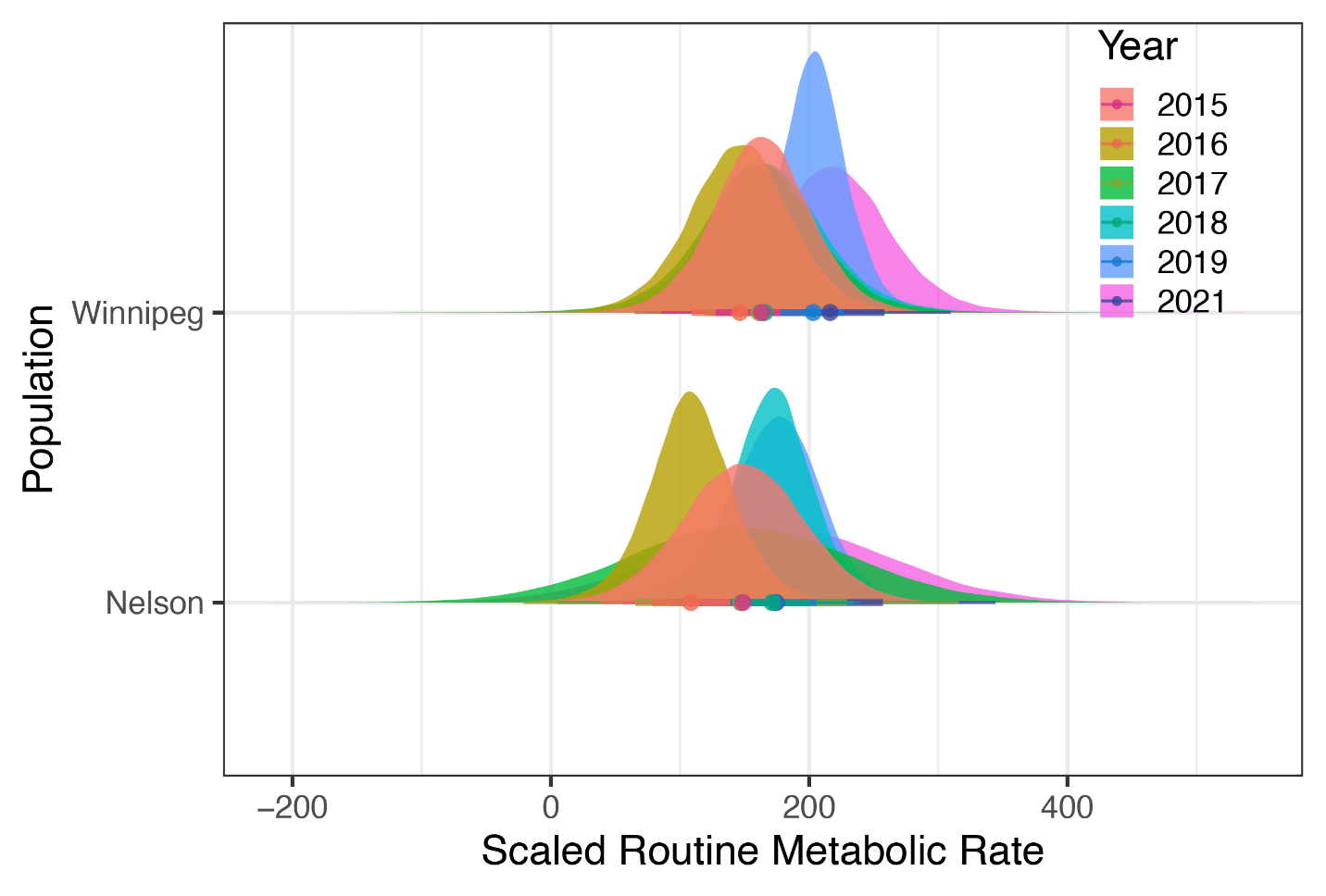


Figure S2. Posterior distributions of scaled routine metabolic rate (mgO_2_Kg^-0.89^Hr^-1^) of age-0 lake sturgeon (*Acipenser fulvescens*) between Winnipeg (southern) and Nelson (northern) population by years. 95% and 66% credible intervals are shown by the thin and bold lines, respectively, below each distribution.


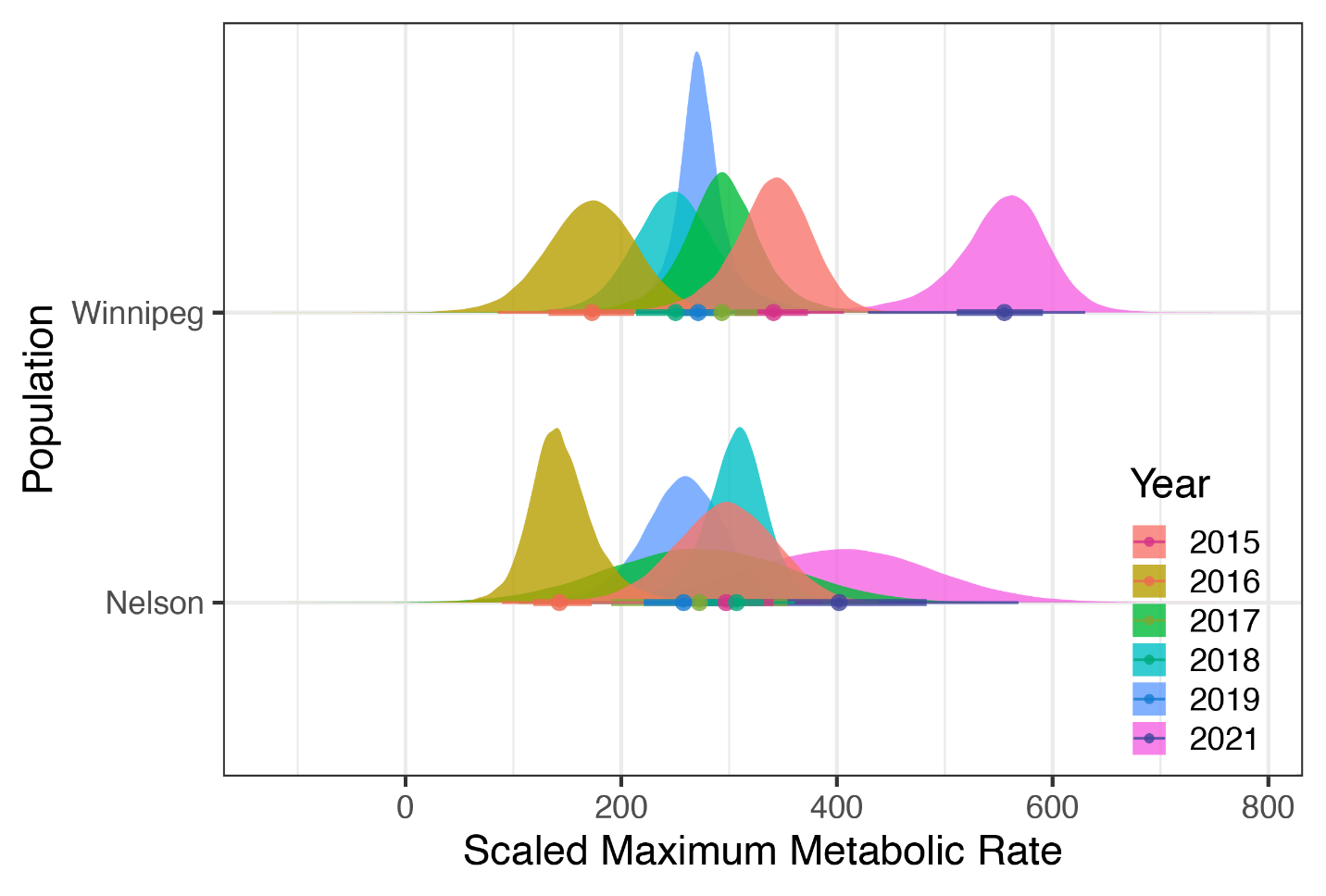


Figure S3. Posterior distributions of scaled maximum metabolic rate (mgO_2_Kg^-0.89^Hr^-1^) of age-0 lake sturgeon (*Acipenser fulvescens*) between Winnipeg (southern) and Nelson (northern) population by years. 95% and 66% credible intervals are shown by the thin and bold lines, respectively, below each distribution.
